# Mechanisms of Anthracycline-Enhanced Reactive Oxygen Metabolism in Tumor Cells

**DOI:** 10.1155/2019/9474823

**Published:** 2019-12-03

**Authors:** James H. Doroshow

**Affiliations:** ^1^Division of Cancer Treatment and Diagnosis, National Cancer Institute, NIH, Bethesda, MD 20892, USA; ^2^Developmental Therapeutics Branch of the Center for Cancer Research, National Cancer Institute, NIH, Bethesda, MD 20892, USA; ^3^Department of Medical Oncology and Therapeutics Research, City of Hope Comprehensive Cancer Center, Duarte, CA 91010, USA

## Abstract

In this investigation, we examined the effect of anthracycline antibiotics on oxygen radical metabolism in Ehrlich tumor cells. In tumor microsomes and nuclei, doxorubicin increased superoxide anion production in a dose-dependent fashion that appeared to follow saturation kinetics; the apparent *K*_m_ and *V*_max_ for superoxide formation by these organelles was 124.9 *μ*M and 22.6 nmol/min/mg, and 103.4 *μ*M and 4.8 nmol/min/mg, respectively. In both tumor microsomes and nuclei, superoxide formation required NADPH as a cofactor, was accompanied by the formation of hydrogen peroxide, and resulted from the transfer of electrons from NADPH to the doxorubicin quinone by NADPH:cytochrome P-450 reductase (NADPH:ferricytochrome oxidoreductase, EC 1.6.2.4). Anthracycline antibiotics also significantly enhanced superoxide anion production by tumor mitochondria with an apparent *K*_m_ and *V*_max_ for doxorubicin of 123.2 *μ*M and 14.7 nmol/min/mg. However, drug-stimulated superoxide production by mitochondria required NADH and was increased by rotenone, suggesting that the proximal portion of the electron transport chain in tumor cells was responsible for reduction of the doxorubicin quinone at this site. The net rate of drug-related oxygen radical production was also determined for intact Ehrlich tumor cells; in this system, treatment with doxorubicin produced a dose-related increase in cyanide-resistant respiration that was enhanced by changes in intracellular reducing equivalents. Finally, we found that in the presence of iron, treatment with doxorubicin significantly increased the production of formaldehyde from dimethyl sulfoxide, an indication that the hydroxyl radical could be produced by intact tumor cells following anthracycline exposure. These experiments suggest that the anthracycline antibiotics are capable of significantly enhancing oxygen radical metabolism in Ehrlich tumor cells at multiple intracellular sites by reactions that could contribute to the cytotoxicity of this class of drugs.

## 1. Introduction

The anthracycline antibiotics, including doxorubicin and daunorubicin, play an important role in the treatment of human leukemias and lymphomas as well as carcinomas of the breast [1]. The major long-term toxicity of anthracycline therapy is a form of cumulative cardiac toxicity that may produce long-lived morbidity, especially in pediatric cancer patients receiving this class of drugs [2]. A substantial body of experimental evidence has been developed suggesting that the cardiac toxicity of the anthracycline antitumor agents may, in part, be related to the generation of strong oxidant species in the heart [3] catalyzed by flavin dehydrogenases present in multiple subcellular compartments [4]. Redox cycling of the anthracycline quinone moiety by complex I of the cardiac electron transport chain [5] can damage intracellular lipid membranes in mitochondria as well as sodium and calcium transporters in the heart [6]. In addition to redox cycling of the quinone functionality of the anthracyclines, doxorubicin may form a potent drug-iron complex that enhances the formation of powerful oxidants with the chemical characteristics of the hydroxyl radical [7, 8]. In this fashion, as well as through interactions with various iron binding proteins, the anthracycline antibiotics may contribute to myocyte toxicity [9, 10].

A role for reactive oxygen species (ROS) produced by redox cycling of the anthracycline quinone in tumor cell killing is also supported by several lines of evidence [11, 12]. These studies include the demonstration by our laboratory that anthracycline-related tumor cell cytotoxicity can be significantly diminished by the administration of reactive oxygen scavengers [13] or by the modification of intracellular antioxidant defenses in vitro and that resistance to tumor cell killing by doxorubicin can be decreased by inhibition of peroxide detoxification [14]. The oxidative metabolism of the anthracyclines has also been demonstrated to occur in vivo; peripheral blood mononuclear cells taken from breast cancer patients treated with doxorubicin reveal the hallmarks of hydroxyl radical damage to DNA in a fashion that is directly related to the length of time during which patients receive the anthracycline [15]. However, unlike studies in the mammalian heart, no comprehensive evaluation of the sites and mechanisms of anthracycline-induced ROS production for mammalian tumor cells has been developed.

Hence, in the current study, we investigated the mechanisms by which anthracycline antibiotics are metabolized to generate ROS in multiple tumor cell compartments, as well as in intact tumor cells. Our results indicate that, in analogy to findings in cardiac subcellular compartments, drugs of the anthracycline class stimulate superoxide anion and hydrogen peroxide production in tumor cell mitochondria, in microsomes, and in the nucleus. Furthermore, in the presence of doxorubicin, extracellular hydrogen peroxide can be quantitated using intact tumor cells. Finally, under appropriate experimental conditions, doxorubicin-induced ROS formation was found to culminate in the production of the hydroxyl radical, or a similar chemical reactant with equivalent oxidizing potency. Thus, drug-related oxygen radical metabolism could pose an important threat to tumor cell integrity by the production of a strong oxidant stress at multiple cellular sites, contributing to cytotoxic membrane injury and DNA damage.

## 2. Materials and Methods

### 2.1. Materials

Daunorubicin, rubidazone, aclacinomycin A, and 5-iminodaunorubicin were supplied by the Drug Synthesis and Chemistry Branch, Division of Cancer Treatment and Diagnosis, National Cancer Institute, Bethesda, MD. All drugs were reconstituted in sterile water on the day of preparation and were protected from light until used. Doxorubicin hydrochloride, glutathione (reduced form), glutathione reductase type III, sodium azide, bovine albumin (fraction V), xanthine, xanthine oxidase (Grade 1), cytochrome c (type VI from horse heart), EDTA, NADPH type III, NADH Grade III, NADP^+^ Grade V, flavin adenine dinucleotide Grade III, flavin mononucleotide, sodium succinate, sucrose, dicumarol, D-mannitol, dimethyl sulfoxide Grade I, EGTA, sodium HEPES, DTNB, rotenone, antimycin A, Triton X-100, urea, sodium benzoate, Tris, and bovine erythrocyte superoxide dismutase (SOD) (2750 units/mg as assayed by the method of McCord and Fridovich [16]) were purchased from Sigma. Methanol (spectral grade), ethyl alcohol (99% pure), potassium cyanide, sodium acetate, acetic anhydride, ferrous sulfate, ferric chloride, formaldehyde (37% *w*/*v*), ammonium acetate, acetic acid, and acetylacetone were obtained from Fisher Chemical Co., Fair Lawn, NJ. Chelex 100 resin (100 to 200 mesh, sodium salt) was purchased from Bio-Rad Laboratories, Richmond, Calif. Catalase of analytical grade (65,000 units/mg protein) was purchased from Boehringer Mannheim Biochemicals, Indianapolis, Ind., and was devoid of SOD activity when assayed by the method of McCord and Fridovich [16]. Diethylurea and dimethylthiourea were purchased from Aldrich. Dulbecco's phosphate buffered saline was obtained from Grand Island Biological Co., Grand Island, NY. BCNU was purchased from Bristol-Myers Squibb. All chemicals were obtained at the highest grade available and were used without further purification. Only glass-distilled, deionized water was used in these studies.

### 2.2. Cell Lines

Ehrlich-Lettré tumor cells (ascitic variant) and P388 leukemia cells were initially obtained from Dr. T. Khwaja of the University of Southern California/Norris Comprehensive Cancer Center and were maintained by weekly passage of one million cells i.p. in 20 g female Swiss-Webster mice. For experiments examining the effect of anthracyclines on cellular oxygen radical production, tumor cells were harvested 5-6 days after implantation, washed twice in 0.9% NaC1, exposed to hypotonic shock lysis to remove contaminating erythrocytes [17], and resuspended in PBS that had been treated with Chelex 100 resin to remove trace quantities of iron from the reagent grade buffer. Cell viability (routinely >95%) was confirmed by exclusion of 0.1% trypan blue dye.

### 2.3. Preparation of Tumor Cell Organelles

To prepare the microsomal fraction, Ehrlich tumor cells were resuspended in 4 volumes of an iced solution of 230 mM Tris-HC1 pH 7.4, containing 1 mM EDTA. Tumor cells were sonically disrupted on ice for 90 sec with six 15 sec bursts, each separated by a 10 sec cooling period, using a VWR Biosonik IV ultrasonicator at a power output of 90 watts. Tumor cells were homogenized further on ice with a Dounce homogenizer using 15 strokes of the tight-fitting pestle, and the microsomal fraction was then prepared by differential ultracentrifugation using the method of Hinnen et al. [18]. The pellet from the final 65,000 x *g* centrifugation step was washed twice and resuspended before use in 150 mM potassium phosphate buffer, pH 7.4, containing 100 *μ*M EDTA.

The tumor cell mitochondrial fraction was prepared in 3 volumes of 0.23 M mannitol, 0.07 M sucrose, 5 mM Tris-HC1, and 1 mM EDTA, pH 7.4 by sonic disruption of cells in mannitol:sucrose:tris:EDTA for 45 sec on ice with subsequent Dounce homogenization as described above. The mitochondrial fraction was then obtained as previously described [19] with resuspension before use in 250 mM sucrose containing 20 mM HEPES, pH 7.

The nuclear fraction was prepared by modification of the method of Mamaril et al. [20]. Cells were washed twice at 4°C in 50 mM KC1 and 50 mM Tris-HC1, pH 7.5, containing 5 mM MgC1_2_ and then resuspended in 6 volumes of iced 10 mM NaC1, 10 mM Tris-HC1, pH 7.4, and 1.5 mM MgC1_2_. Ehrlich cells were then centrifuged at 10,000 x *g* for 10 min at 4°C, resuspended in 8 volumes of the NaC1:tris:MgC1_2_ buffer, and disrupted with 15 strokes of the Dounce homogenizer. Cells were homogenized further on ice with several 15 sec bursts of the ultrasonicator. The degree of cell disruption was checked after each sonic burst by phase contrast microscopy; sonication was continued until at least 95% of the tumor outer membranes had been broken. The cell suspension was then centrifuged at 600 x *g* and 4°C for 10 min; the supernatant was discarded and the pellet was resuspended in 2 volumes of 220 mM sucrose, 9.4 mM KH_2_PO_4_, 12.5 mM KH_2_PO_4_, 10 mM MgC1_2_, 2 mM EDTA, and 300 *μ*M NaHCO_3_, pH 7.0. The nuclear suspension was freed of excess cytoplasm with 10-20 strokes of the Dounce homogenizer and then added on ice to 7 volumes of 2.3 M sucrose with stirring. The purified nuclear fraction was prepared using a discontinuous sucrose gradient when 1 ml of 2.3 M sucrose solution containing nuclei was underlaid with 2 ml of 2.1 M sucrose and then centrifuged at 4°C and 105,000 x *g* for 20 min. The nuclear layer was resuspended in 250 mM sucrose and 20 mM HEPES, pH 7.4, before use. The nuclear fraction prepared in this manner was examined for contamination by extranuclear membranes by measurement of marker enzymes and by phase contrast microscopy [20]. The Ehrlich tumor nuclei used in this study contained <10% of the specific glutamate dehydrogenase and cytochrome oxidase activities of the initial cell homogenate and were essentially free of extranuclear membranes by microscopic methods. The experimental microsomal, mitochondrial, and nuclear fractions were studied on the day of preparation.

### 2.4. Measurement of Superoxide Anion Production and NAD(P)H Consumption

Superoxide anion production in experimental samples was determined by the rate of SOD-inhibitable acetylated cytochrome c reduction as previously described [4]. The initial, linear rate of acetylated cytochrome c reduction was determined spectrophotometrically at 550 nm and 37°C in a Gilford spectrophotometer equipped with a circulating water bath. For experiments assessing the effect of DTNB on superoxide production, the sulfhydryl reagent was added to the paired reaction mixtures which were then preincubated for 2 min at 37°C before the addition of NADPH. Preincubation was not performed in experiments examining the effect of other agents on the rate of superoxide formation. Specific conditions for measurement of superoxide production by drug-treated microsomal, mitochondrial, and nuclear fractions have been described in the legends for the appropriate tables.

The effect of anthracycline antibiotics on the oxidation of NAD(P)H by subcellular fractions from Ehrlich cells was determined in triplicate at 37°C by the linear decrease in absorbance at 340 nm. NAD(P)H consumption was initiated by the addition of the membrane protein and was calculated using an extinction coefficient of 6.22 nM^−1^ cm^−1^ [21].

### 2.5. Measurement of Oxygen Consumption and Hydrogen Peroxide Formation

The rate of oxygen consumption by Ehrlich tumor cells was determined at 37°C with a Model 53 oxygen monitoring system (Yellow Springs Instrument Co., Yellow Springs, Ohio). The 3 ml reaction system usually contained 15 × 10^6^ cells and PBS that had been bubbled with air for 30 min at 37° before use. When KCN, rotenone, antimycin A, glucose, or BCNU were added to this system, they were preincubated with the cells for 5 min at 37°. Oxygen consumption by subcellular fractions was determined in a similar fashion after equilibration of the membrane preparation and drugs with the particular buffer used for 4 min in the reaction vessel; these reactions were initiated by addition of appropriate cofactors, usually NAD(P)H. Free radical scavengers, when used, were added to the reaction vessel and equilibrated for 5 min prior to the addition of the anthracycline antibiotic. The linear rate of oxygen consumption was determined from 10 to 30 min after drug treatment. Hydrogen peroxide production was quantitated by the release of oxygen into the system as previously described [22] after the addition of 10 *μ*l of catalase (4500-9000 units) through the access slot of the oxygen electrode plunger. The rate of oxygen consumption was calculated from a value of 597 nmol for the total dissolved oxygen of the reaction mixture [23].

### 2.6. Measurement of Hydroxyl Radical Formation

The formation of ^·^OH, or an oxidizing species with the chemical reactivity of ^·^OH, by permeabilized Ehrlich cells treated with doxorubicin was assessed by measurement of formaldehyde production from dimethyl sulfoxide (DMSO) as described by Klein et al. [24]. The standard reaction mixture contained 100 mM DMSO, 100 *μ*M Na_4_EDTA, 1 mM NADPH, 0.1% (*v*/*v*) Triton X-100, 1 × 10^7^ cells/ml, and the indicated amount of doxorubicin in a final volume of 7 ml of PBS at pH 7.2. Iron-EDTA was added to the reaction systems as a 1 : 2 mixture of freshly prepared ferrous sulfate in aqueous Na_4_EDTA. Quantitation of ^·^OH production in this system was undertaken because of our interest in determining whether or not enrichment of flavin dehydrogenase-specific activities by preparation of subcellular fractions was necessary to demonstrate oxy-radical production (other than O_2_ consumption and H_2_O_2_ formation) by drug-treated tumor cells.

The reaction systems were initiated by the addition of NADPH; they were then mixed vigorously and incubated, usually for 2 hr at 37°C, in 25 ml polycarbonate flasks in a shaking water bath. The reactions were terminated by the addition of 0.5 ml of ice-cold 17.5% (*w*/*v*) trichloroacetic acid to 1 ml aliquots taken from the standard reaction mixture. Samples were centrifuged at 1000 x *g* for 10 min in the cold; a 1 ml portion of the supernatant was then assayed for formaldehyde [24, 25]. In brief, 1 ml of a solution containing 2 M ammonium acetate, 50 mM acetic acid, and 20 mM acetylacetone was added to the 1 ml experimental sample; the sample was then mixed, incubated at 37°C for 40 min in a shaking water bath, and assayed for relative formaldehyde concentration at 25°C in a 1 ml volume by spectrophotometric measurement at 410 nm. Zero-time samples, as well as samples lacking the reduced pyridine nucleotide cofactor, DMSO, or Ehrlich cells were routinely used as the blanks.

Preliminary experiments revealed that in our assay system, doxorubicin itself at a concentration of 300 *μ*M did not significantly affect the quantitation of defined amounts of authentic formaldehyde. We also examined the effect of Ehrlich cells on the recovery of formaldehyde added to the experimental reaction mixture. Compared to a cell-free system, we found that over the concentration range from 10 to 70 *μ*M, the recovery of genuine formaldehyde varied from 100 to 89% in the presence of 10^7^ Ehrlich cells/ml.

Formaldehyde levels were determined from a calibration curve that was linear at concentrations from 10 to 250 *μ*M; the calibration curve was routinely prepared in the standard reaction mixture of each experimental condition without cells or doxorubicin and was processed as described above for each set of experimental samples. The lower limit of sensitivity for the detection of formaldehyde in this assay was approximately 10 *μ*M.

### 2.7. Enzyme Assays

The NADPH:cytochrome P-450 reductase activity of the tumor microsomal and nuclear fractions was measured by a technique described previously [4] using nonacetylated cytochrome c as the electron acceptor. To assess the effect of DTNB (100 *μ*M) and NADP^+^ (1 mM) on NADPH:cytochrome P-450 reductase activity, these reagents were preincubated with the fraction for 2 min before the addition of NADPH. Glutathione peroxidase activity was determined in tumor cell subcellular fractions as described previously [26] except that enzyme assays were initiated with 440 rather than 220 nmol hydrogen peroxide in these experiments. The data have been expressed as nmol NADPH oxidized to NAPD^+^ per min mg protein. SOD levels were determined in the microsomal, mitochondrial, and nuclear fractions using the xanthine:xanthine oxidase:cytochrome c assay as reported previously [26]. In these experiments, acetylated cytochrome c (11.2 *μ*M) was utilized in addition to KCN (10 *μ*M) to eliminate interference from cytochrome oxidases in the experimental samples.

### 2.8. Protein Determination

Protein concentrations in subcellular fractions were determined by the method of Lowry et al. [27] using crystalline bovine albumin as the standard.

### 2.9. Statistical Methods

Data were analyzed with the 2-tailed *t* test for independent means (not significant, *P* > 0.05 [28]).

## 3. Results

### 3.1. Tumor Microsomes

As shown in Table 1, treatment with doxorubicin increased microsomal superoxide production in a dose-dependent fashion that appeared to conform to saturation kinetics. Furthermore, a doxorubicin concentration as low as 5 *μ*M significantly increased oxy-radical formation over control levels (data not shown). In these experiments, superoxide production varied with the amount of microsomal protein used. In the absence of doxorubicin, superoxide formation was (mean ± S.E.; *n* = 3) 0.42 ± 0.10 and 0.96 ± 0.20 nmol/min with 100 and 400 *μ*g protein/ml, respectively; in the presence of doxorubicin (135 *μ*M), superoxide production was 1.73 ± 0.07 and 3.14 ± 0.08 nmol/min, *P* < 0.01 for each compared to control. As shown in Table 2, all components of the reaction system, including NADPH, intact microsomes, and acetylated cytochrome c were necessary to demonstrate a significant increase in superoxide formation by doxorubicin. Furthermore, only NADPH, of a variety of cofactors, could support drug-related superoxide production in these studies (Table 2).

The specificity of our assay for drug-stimulated superoxide production was also addressed (Table 2). We found that SOD-inhibitable cytochrome c reduction was not affected by catalase or DMSO in concentrations capable of eliminating either H_2_O_2_ or the hydroxyl radical from the reaction system (Table 2). The addition of heat-denatured SOD to the experimental system also produced no significant change in the rate of drug-stimulated superoxide production. These experiments suggest that superoxide was, in fact, measured in our studies.

As shown in Table 3, three of the four anthracycline antibiotics tested in addition to doxorubicin significantly increased microsomal superoxide production over control levels. However, 5-iminodaunorubicin, an anthracycline analog that has been previously demonstrated under other conditions to be incapable of redox cycling because of its substituted quinone ring [4], did not stimulate superoxide production by the tumor microsomal fraction.

To define the mechanism of oxygen radical production by anthracycline-treated microsomes, we measured the rate of NADPH consumption in tumor microsomes after treatment with doxorubicin. In the presence of 100 *μ*M NADPH and 100 *μ*g/ml of microsomal protein from tumor, the control rate of NADPH oxidation was 7.00 ± 0.81 nmol/min/mg, *n* = 4; the addition of doxorubicin (135 *μ*M) increased the rate of NADPH oxidation to 19.90 ± 2.30 nmol/min/mg, *n* = 4, *P* < 0.01. Furthermore, we found that the tumor microsomal fraction contained a substantial level of NADPH:cytochrome P-450 reductase activity (Table 4). After treatment of microsomes with the enzyme inhibitors DTNB or excess NADP^+^, enzyme activity decreased to 27.7 or 23.2% of control levels, *P* < 0.01 for both agents (Table 4). In parallel experiments, DTNB or excess NADP^+^ decreased drug-related superoxide production to 41.3 or 26.6% of baseline levels, *P* < 0.01 for each inhibitor (Table 4). Finally, we found that the inhibitor of NADPH:quinone oxidoreductase 1, dicumarol, produced no significant effect on doxorubicin-stimulated superoxide production (Table 2), suggesting that this quinone reductase is not involved in the metabolism of anthracyclines by tumor microsomes. Taken together, these experiments strongly suggest that the NADPH:cytochrome P-450 reductase activity of the Ehrlich tumor microsomal fraction is responsible for the one-electron reduction of anthracycline antibiotics at this site.

**Figure 1:**
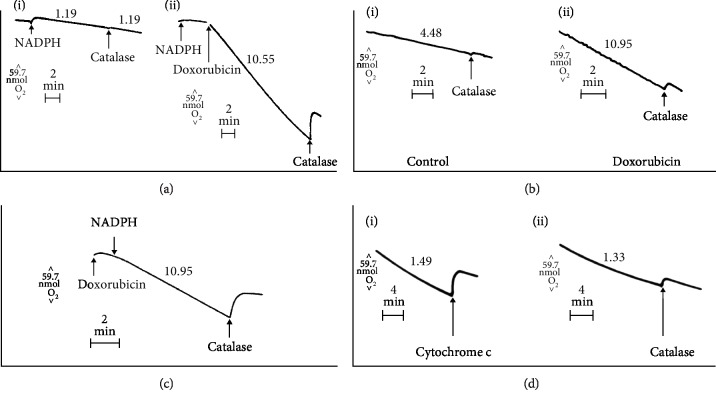
Effect of doxorubicin on oxygen consumption by Ehrlich tumor organelles. Oxygen consumption in tumor microsomes was examined as described in Supplementary [Supplementary-material supplementary-material-1]. (a, i) is without drug; (ii) shows the results with doxorubicin. The addition of NADPH (1 mM), catalase (4500 units), or doxorubicin (135 *μ*M) to the 3 ml vessel was performed through the access slot of the oxygen electrode and has been indicated (arrow). The numbers above each tracing indicate the rate of oxygen consumption (nmol/min/mg). (b) Effect of doxorubicin on oxygen consumption by Ehrlich tumor mitochondria. Oxygen consumption in these representative experiments was performed as described in Supplementary [Supplementary-material supplementary-material-1]. (i) is the control reaction; (ii) represents the identical experiment in the presence of doxorubicin (135 *μ*M). The addition of catalase (4500 units) to the 3 ml vessel was performed through the access slot of the oxygen electrode and has been indicated by the arrow. The numbers above each tracing indicate the rate of oxygen consumption (nmol/min/mg). (c) Effect of doxorubicin on oxygen consumption by Ehrlich tumor nuclei. Experimental conditions consisted of a 3 ml system containing 250 mM sucrose, 20 mM HEPES, pH 7.4, 100 *μ*M EDTA, 1 mM NADPH, and 200 *μ*g/ml of nuclear protein at 37°C. The addition of NADPH (1 mM), doxorubicin (135 *μ*M), or catalase (4500 units) to the 3 ml reaction vessel was performed through the access slot of the oxygen electrode and has been indicated by an arrow. The number above the tracing is the rate of oxygen consumption (nmol/min/mg). (d) Doxorubicin-stimulated oxygen consumption by Ehrlich tumor cells. The effect of doxorubicin (400 *μ*M) on oxygen consumption by Ehrlich cells (5 × 10^6^ cells/ml) is shown in representative examples from multiple experiments. The addition of acetylated cytochrome c (168 nmol) in (i) or catalase (9000 units) in (ii) to the 3 ml vessel has been indicated by an arrow. The numbers above each tracing indicate the rate of oxygen consumption (nmol/min/ml).

To confirm that anthracycline antibiotics increased reactive oxygen production in tumor microsomes, we examined the effect of doxorubicin on microsomal oxygen consumption. As demonstrated in Supplementary [Supplementary-material supplementary-material-1], oxygen consumption increased more than 6-fold in the presence of doxorubicin, *P* < 0.001. Drug-stimulated oxygen consumption was significantly inhibited by cytochrome c which reacts directly with superoxide to return oxygen to this closed system and was significantly increased by KCN, probably because of the inhibition of microsomal SOD (Table 5). As shown in Figure 1(a), we found that oxygen was released routinely by addition of excess catalase to microsomes treated with doxorubicin, indicating that H_2_O_2_ as well as superoxide anion had been produced in these investigations. We found that H_2_O_2_ production increased from undetectable control levels (*n* = 3) to 3.42 ± 0.48 nmol/min/mg (*n* = 4; *P* < 0.01) after treatment of the microsomal fraction with doxorubicin (135 *μ*M). Thus, NADPH-dependent microsomal metabolism of doxorubicin results in both superoxide anion and H_2_O_2_ formation.

### 3.2. Tumor Mitochondria

Because previous studies in cardiac tissue had indicated that the electron transport chain could reduce doxorubicin to its semiquinone [4], we examined the effect of doxorubicin on reactive oxygen formation by the Ehrlich tumor mitochondrial fraction. We found that for tumor mitochondria, as well as microsomes, treatment with doxorubicin increased superoxide formation in a dose-dependent fashion that also appeared to follow saturation kinetics (Table 1). However, in mitochondria, superoxide production was NADH- rather than NADPH-dependent and was significantly increased by addition of rotenone to block electron flow beyond complex I of the electron transport chain (Table 6). Furthermore, substituting NAD^+^ (100 *μ*M), NADP^+^ (100 *μ*M), or succinate (1 mM) for NADH yielded no detectable drug-enhanced superoxide production by tumor mitochondria (data not shown).

Superoxide production in the mitochondrial fraction after treatment with doxorubicin (135 *μ*M) increased from (*n* = 3) 0.37 ± 0.03 to 0.76 ± 0.02 and 1.05 ± 0.07 nmol/min when 50, 150, or 200 *μ*g/ml of mitochondrial protein was employed. In contrast, superoxide production by tumor mitochondria in the absence of doxorubicin was 0.10 ± 0.04, 0.18 ± 0.05, and 0.21 ± 0.03 nmol/min for the identical levels of mitochondrial protein, *P* < 0.01 for each drug-treated sample compared to control. We also found that the anthracycline antibiotics which had significantly enhanced microsomal superoxide production also increased mitochondrial oxy-radical formation (Table 6). Finally, we found that doxorubicin (135 *μ*M) stimulated the rate of NADH oxidation by tumor mitochondria (100 *μ*g/ml) in the presence of rotenone from 4.70 ± 0.42 (*n* = 3) to 9.74 ± 0.63 (*n* = 3) nmol/min/mg, *P* < 0.01. Thus, it is likely that an early portion of the NADH dehydrogenase complex is responsible for the reduction of anthracycline antibiotics to free radicals in tumor mitochondria.

To investigate the effect of doxorubicin on mitochondrial reactive oxygen metabolism further, the rate of oxygen consumption by drug-treated mitochondria was examined. As shown in Supplementary [Supplementary-material supplementary-material-1], doxorubicin significantly increased the rate of mitochondrial oxygen consumption; furthermore, the rate of drug-stimulated oxygen consumption was not affected by KCN or dicumarol, suggesting that (1) mitochondrial SOD in Ehrlich tumor cells is not inhibited by cyanide and that (2) NADPH:quinone oxidoreductase 1 does not appear to be involved in the free radical metabolism of doxorubicin in tumor mitochondria. On the other hand, drug-stimulated oxygen consumption was decreased to control levels in the presence of acetylated cytochrome c suggesting that most, if not all, of the oxygen consumed had at least initially been converted to superoxide anion (Supplementary [Supplementary-material supplementary-material-1]). We also found (Figure 1(b)) that treatment with doxorubicin increased mitochondrial H_2_O_2_ production from undetectable control levels (*n* = 3) to 2.6 ± 0.6 nmol/min/mg, *n* = 3, *P* < 0.01. Thus, exposure of tumor mitochondria, as well as microsomes, to doxorubicin increases both superoxide anion and hydrogen peroxide production.

### 3.3. Tumor Nuclei

Treatment of Ehrlich tumor nuclei with doxorubicin significantly increased superoxide production (Table 7). In this organelle, oxy-radical formation required intact nuclear protein and NADPH; neither NADH, FAD, nor FMN could support drug-stimulated nuclear superoxide formation. Furthermore, daunorubicin, rubidazone, and aclacinomycin A all significantly increased reactive oxygen metabolism compared to control (Table 7). As shown in Table 1, superoxide production in the nuclear fraction also appeared to follow saturation kinetics. We found that doxorubicin (135 *μ*M) increased the rate of NADPH (100 *μ*M) oxidation by the nuclear fraction (200 *μ*g protein/ml) from a control rate of 2.20 ± 0.31 (*n* = 3) to 11.11 ± 0.73 nmol/min/mg, *n* = 3, *P* < 0.01.

As shown in Supplementary [Supplementary-material supplementary-material-1], Ehrlich tumor nuclei possessed approximately 2% of the NADPH:cytochrome P-450 reductase activity of the microsomal fraction. This enzymatic activity could be inhibited by excess NADP^+^ and could be removed completely by treatment of the nuclear fraction with a nonionic detergent capable of stripping the outer nuclear envelope from underlying DNA and chromatin. In related experiments, we found that doxorubicin-stimulated nuclear superoxide formation could also be significantly inhibited or abolished by treatment of the nuclei with the detergent (Supplementary [Supplementary-material supplementary-material-1]). These experiments strongly suggest that NADPH:cytochrome P-450 reductase activity normally associated with the outer nuclear envelope is responsible for activating doxorubicin to its free radical at that locale.

In analogy to our microsomal and mitochondrial experiments, we investigated nuclear oxygen consumption in the presence of doxorubicin. In this setting, control nuclei consumed 0.93 ± 0.12 nmol O_2_/min/mg, *n* = 3. Oxygen consumption increased 10-fold in the presence of doxorubicin (135 *μ*M) to 9.45 ± 0.89 nmol/min/mg, *n* = 6, *P* < 0.01. Treatment with KCN (1 mM) did not increase drug-stimulated oxygen consumption significantly (11.04 ± 0.80 nmol/min/mg, *n* = 4), probably because of the small amount of SOD associated with the nuclear fraction (Table 5). However, the addition of acetylated cytochrome c (56 *μ*M) decreased oxygen consumption to 2.09 ± 0.26 nmol/min/mg, *n* = 3, *P* < 0.01 compared to samples containing doxorubicin alone. Thus, most of the oxygen consumed had probably been reduced, at least initially, to superoxide anion. As shown in Figure 1(c), drug-enhanced oxygen consumption required NADPH and was associated with the production of H_2_O_2_. In these studies, drug treatment (135 *μ*M) increased H_2_O_2_ formation from undetectable control levels (*n* = 3) to 5.2 ± 1.3 nmol/min/mg, *n* = 3, *P* < 0.01.

### 3.4. Whole Tumor Cells

**Figure 2:**
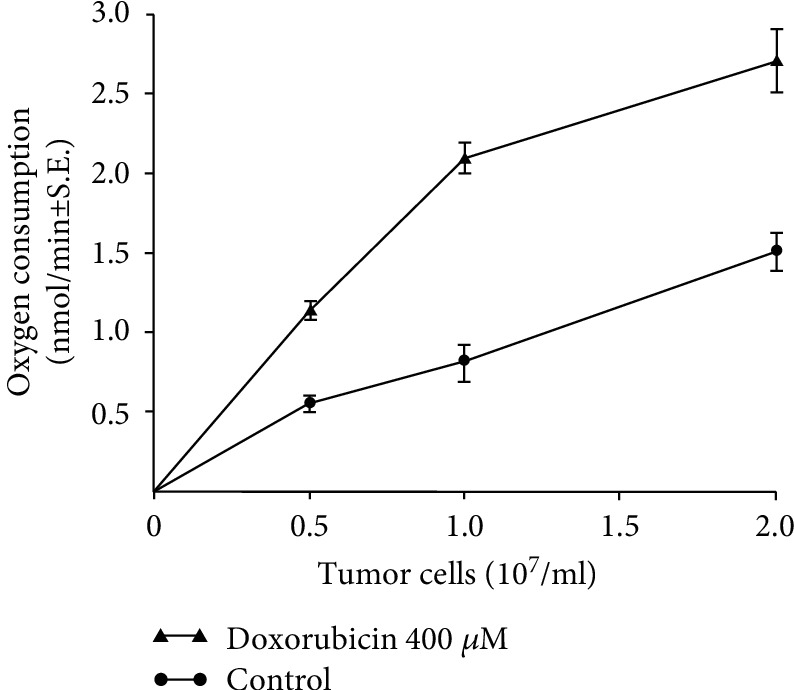
Effect of tumor cell number on the rate of cyanide-resistant oxygen consumption in the presence and absence of doxorubicin. The doxorubicin concentration used for these experiments was 400 *μ*M. These studies were performed as described in Table 8; the data represent the mean ± S.E. of 3 determinations at each tumor cell concentration.

To examine the net effect of anthracycline antibiotics on oxy-radical production by Ehrlich tumor, we investigated whether doxorubicin altered the respiratory rate of intact tumor cells. Treatment with doxorubicin significantly increased the rate of oxygen consumption by tumor cells in which the electron transport chain had been blocked by either cyanide or antimycin A (Table 8) [29, 30]. It is likely that the magnitude of the overall rate of mitochondrial O_2_ consumption in tumor cells makes the smaller, drug-related effects impossible to detect in the absence of respiratory chain blockade. We found that the increase in respiratory rate was drug-dose dependent and was found for daunorubicin as well as doxorubicin; however, the daunorubicin analog 5-iminodaunorubicin which has a blocked quinone group that does not permit redox cycling did not enhance cyanide-resistant respiration by Ehrlich cells [31, 32]. In control experiments, we found that tumor cell viability in the presence of KCN as measured by exclusion of trypan blue dye did not decline significantly over the reaction period of these studies. Furthermore, the addition of glucose to the cells, which produces an increase in intracellular NADPH concentration [22], significantly increased the rate of O_2_ consumption after doxorubicin administration (Table 8). We found that alterations in osmolality by glucose did not explain these observations, since when buffers of identical osmolality were used (with or without glucose), the rate of O_2_ consumption was significantly higher only in the presence of glucose (data not shown). As shown in Figure 2, cyanide-resistant respiration in the presence or absence of doxorubicin varied with the number of cells used in the experiment; however, the rate of oxygen consumption was always significantly higher (*P* < 0.01) when the drug was present. Finally, when experiments identical to those in Table 8 were performed with P388 murine leukemia cells treated with KCN, we found that doxorubicin (400 *μ*M) increased O_2_ consumption from (mean ± S.E.; *n* = 3) the 0.31 ± 0.05 control rate to 1.15 ± 0.08 nmol/min/5 × 10^6^ cells, *P* < 0.01. Taken together, these experiments suggest that anthracycline antibiotics enhance oxy-radical production by tumor cells in a process that requires an intact quinone ring and may be modulated by the supply of cellular reducing equivalents. These features are similar to those previously described for the tumor cell organelles examined in this study; they reveal, further, that anthracycline redox cycling may overcome the ability of intact tumor cells to detoxify ROS.

As shown in Figure 1(d), the addition of cytochrome c or catalase to Ehrlich cells treated with doxorubicin (400 *μ*M) led to the release of oxygen in this closed system, indicating that H_2_O_2_ had been formed. We found that in the presence of doxorubicin (400 *μ*M), Ehrlich cells produced 0.64 ± 0.04 nmol H_2_O_2_/min/10^7^ cells compared to undetectable levels of hydrogen peroxide in the absence of the drug, *P* < 0.01, *n* = 3. Because the tumor cell outer membrane is impermeable to high molecular weight proteins such as catalase, this estimate of H_2_O_2_ formation probably reflects only the proportion of the total H_2_O_2_ pool present extracellularly; thus, it is not possible to make a direct stoichiometric comparison between total cyanide-resistant O_2_ consumption and H_2_O_2_ formation, even under identical experimental conditions.

Oxy-radical cascades, which include the production of H_2_O_2_, may also be capable of supporting the formation of the potent oxidizing radical ^·^OH, or a related molecule with similar chemical reactivity [33]. We investigated the mechanism of hydroxyl radical formation by Ehrlich tumor cells after treatment with doxorubicin by quantitation of the formaldehyde produced in the reaction of DMSO with ^·^OH. As previously demonstrated, on a molar basis, formaldehyde is the major by-product of this reaction between the reactive oxygen metabolite and DMSO [24]. We maximized the possibility of measuring drug-enhanced ^·^OH production in these experiments by utilizing a nonionic detergent to increase the access of both doxorubicin and pyridine nucleotide cofactors to intracellular or membrane-bound dehydrogenases. The requirements for formaldehyde production by Ehrlich carcinoma cells are shown in Table 9; the hydroxyl radical was detected in this system only in the presence of the anthracycline antibiotic. Furthermore, drug-stimulated formaldehyde production was measured only when the tumor cells, DMSO, iron-EDTA, and Triton X-100 were all present in these experiments. Increasing the FeSO_4_ concentration above 50 *μ*M did not enhance formaldehyde production further (data not shown); whereas, FeSO_4_ levels as low as 1 *μ*M still supported substantial formaldehyde formation (Table 9). Furthermore, FeC1_3_ at a concentration of 50 *μ*M was also capable of supporting doxorubicin-enhanced formaldehyde production by Ehrlich cells; thus, both ferric and ferrous iron may serve as catalysts for this process (Table 9). In these studies, both NADPH and NADH, but not succinate, could provide the reducing equivalents necessary for oxygen radical metabolism in Ehrlich cells treated with doxorubicin. These cofactor requirements are consistent with the previously presented experiments demonstrating that the microsomal, mitochondrial, and nuclear fractions from Ehrlich cells contain different NADPH- and NADH-dependent dehydrogenases capable of catalyzing the reduction of doxorubicin to its semiquinone free radical intermediate [34–37].

**Figure 3:**
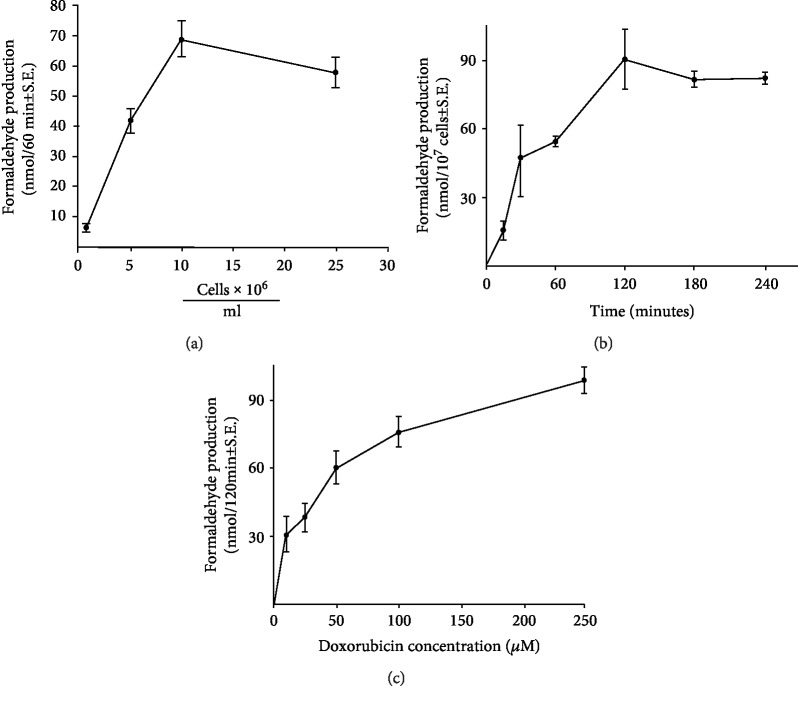
Effect of Ehrlich tumor cell number on doxorubicin-stimulated formaldehyde production. (a) Formaldehyde production from DMSO was assessed spectrophotometrically in the presence of a fixed concentration of doxorubicin (250 *μ*M) over a 60 min period of incubation; the data represent the mean ± S.E. of three experiments for each concentration of tumor cells. (b) Effect of the duration of incubation on doxorubicin-induced formaldehyde formation by Ehrlich tumor cells. The extent of formaldehyde production by Ehrlich cells (10^7^/ml) in the presence of doxorubicin (250 *μ*M) was determined as a function of the time after initiation of the reaction. Each time point represents the mean ± S.E. of three experiments. (c) Effect of doxorubicin concentration on formaldehyde production by Ehrlich tumor cells. In these studies, the production of formaldehyde from DMSO was examined at a tumor cell concentration of 10^7^/ml; the data represent the mean ± S.E. of three experiments at each doxorubicin level tested.

Following the determination that doxorubicin could stimulate formaldehyde production from DMSO by Ehrlich cells, we examined several characteristics of ^·^OH formation in this system. As shown in Figure 3(a), formaldehyde production by the carcinoma cells varied with the concentration of tumor cells used in the assay; at a doxorubicin level of 250 *μ*M, using a 60 min incubation time, peak formaldehyde production (mean ± S.E.; *n* = 3; 69.5 ± 6 nmol) occurred with 10^7^ tumor cells/ml in the reaction mixture. We found that under similar experimental conditions, the amount of formaldehyde produced was also related to the concentration of DMSO used for the study; formaldehyde formation (mean ± S.E., *n* = 3) increased from 33.2 ± 6.1 nmol/60 min/10^7^ cells to 37.1 ± 7.2, 56.9 ± 3.4, 59.7 ± 26.1, and 47.1 ± 6.3 when the DMSO concentration varied from 5 to 50, 100, 200, or 1000 mM. Thus, a DMSO concentration of 100 mM was chosen for all subsequent experiments. When the reaction interval was altered at a fixed dose of doxorubicin (Figure 3(b)), formaldehyde formation increased from undetectable levels at the zero-time point to 90.2 ± 16.4 nmol/10^7^ cells, *n* = 3, at 2 hr; no further, significant increase in hydroxyl radical production could be demonstrated when the incubation time was extended for up to 4 hr after the initiation of the reaction (Figure 3(b)).

Doxorubicin increased hydroxyl radical production by Ehrlich carcinoma cells in a dose-related fashion over a wide range of drug concentrations (Figure 3(c)). Drug-stimulated formaldehyde production under our experimental conditions could be reproducibly measured after treatment of the cells with a doxorubicin concentration as low as 5 *μ*M (24.3 ± 11.8 nmol/120 min/10^7^ cells, *n* = 3).

To examine the mechanism of formaldehyde production and to verify that the evolution of formaldehyde from DMSO was a measurement of ^·^OH formation, we investigated the effect of various oxygen radical scavengers on the level of formaldehyde produced by treatment of Ehrlich cells with doxorubicin. The addition of SOD or catalase, but not the heat-inactivated enzymes, significantly decreased or abolished drug-related formaldehyde production (Table 10). These results suggested that both the superoxide anion and hydrogen peroxide were necessary for the generation of ^·^OH by Ehrlich carcinoma cells. We also found that sodium benzoate, mannitol, diethylurea, and dimethylthiourea, which are all potent scavengers of the hydroxyl radical [38], were between 31 and 100% effective in competing with DMSO for reaction with ^·^OH. However, urea, a structurally similar but ineffective ^·^OH scavenger, had no significant effect on formaldehyde production from DMSO (Table 10). Furthermore, we found that treatment of the permeabilized Ehrlich cells with the ^·^OH scavengers outlined in Table 10 decreased the production of methane, an alternate by-product of the reaction between ^·^OH and DMSO, to the same extent as that found when formaldehyde was assayed (data not shown). This suggests that the hydroxyl radical scavengers actually combined with the oxidant species formed in our experimental system rather than altering the stoichiometry of the reaction pathways involved in the degradation of DMSO by the hydroxyl radical. These results, which indicated that formaldehyde production after treatment of Ehrlich carcinoma cells with doxorubicin was dependent upon the presence of the superoxide anion, hydrogen peroxide, and an iron-EDTA complex, strongly suggest that the hydroxyl radical or a related species with similar reactivity was formed in our experiments.

### 3.5. Antioxidant Levels

To understand the significance of drug-stimulated oxygen radical formation in each subcellular fraction, we determined the glutathione peroxidase and SOD-specific activities associated with these fractions (Table 5). As seen in Table 5, the glutathione peroxidase activity in tumor microsomes and nuclei is approximately 10% or 5% of that in the cytosol. Since Ehrlich cells contain minimal catalase activity [39], glutathione peroxidase is the major cellular defense against hydrogen peroxide. Furthermore, the glutathione peroxidase level of tumor cytosol, while more than 20-fold greater than tumor nuclei, is only 30% as high as the enzyme level in rat heart cytosol [4]. Although the specific activity of glutathione peroxidase in tumor mitochondria exposed to ultrasonic disruption was 26.2% of that in the cytosol, ultracentrifugation of the sonicated mitochondria produced a supernatant with essentially the same specific activity as the tumor cytosol. Thus, it seems very likely that most, if not all, of the mitochondrial glutathione peroxidase is located in the mitochondrial matrix.

As shown in Table 5, the majority of tumor cell SOD is located in the cytosol, with much smaller specific activities associated with the microsomal or nuclear fractions. Mitochondrial SOD was intermediate in specific activity between cytosol and nuclei and appeared to be present in at least two mitochondrial sites. Overall, these experiments suggest that tumor nuclei and microsomes are the least well-protected subcellular fractions with respect to the available antioxidant defenses capable of detoxifying a drug-induced, oxy-radical cascade.

## 4. Discussion

In these experiments, we have provided a comprehensive examination of the sites and mechanisms of anthracycline-stimulated oxy-radical production by Ehrlich carcinoma cells. We found that the microsomal, mitochondrial, and nuclear tumor fractions were each capable of supporting drug-induced superoxide anion and hydrogen peroxide production under appropriate experimental conditions. For tumor microsomes and nuclei, this appeared to be an NADPH-dependent process that resulted from reduction of the anthracycline quinone by the NADPH:cytochrome P-450 reductase activity associated with either the microsomal membrane or the outer nuclear envelope. Mitochondrial anthracycline metabolism, on the other hand, was NADH-dependent and was stimulated by rotenone, suggesting that an early portion of the mitochondrial NADH dehydrogenase complex was responsible for reduction of the anthracycline quinone at this intracellular location in tumor cells. Generation of ROS by anthracycline antibiotics occurred at drug concentrations that are found intracellularly following exposure of intact cells to this class of anticancer agents [40]. These experiments are among the first to demonstrate that anthracycline-related ROS production can occur at essentially every intracellular site in tumor cells leading to the formation of an extracellular, and potentially damaging, peroxide flux.

We also determined that each anthracycline tested, except for the quinone-substituted drug 5-iminodaunorubicin, was capable of enhancing oxy-radical metabolism by every subcellular fraction. Furthermore, we found that reactive oxygen metabolism occurred despite the presence of both SOD and glutathione peroxidase in the tumor organelles. Thus, if these subcellular fractions possess a similar distribution of antioxidant enzymatic defenses in vivo, oxygen radical production by the anthracycline antibiotics might exceed the detoxifying capacity of various tumor cell compartments.

Using intact Ehrlich cells, the anthracycline antibiotics were shown to increase the rate of either cyanide- or antimycin A-resistant respiration. This strongly suggests that doxorubicin and other anthracycline quinones actually undergo the previously described oxidation-reduction reactions in vivo. These experiments are also important because they suggest that manipulation of the intracellular reducing environment, by glucose or by the glutathione reductase inhibitor BCNU (which decreases peroxide detoxification by the GSH-GSH peroxidase cycle), may affect the redox metabolism of doxorubicin in whole tumor cells.

We also determined that treatment of intact cells with doxorubicin was associated with H_2_O_2_ production; furthermore, our experiments suggest that following drug treatment H_2_O_2_ accumulates extracellularly. Thus, the plasma membrane, as well as tumor nuclei, mitochondria, and microsomes, may be at risk from a free radical attack which, in this case, could come from both inside and outside the cell. It is likely that the presence of extracellular H_2_O_2_ is due to either passive transport of H_2_O_2_, produced at several intracellular sites, across the plasma membrane or transport through an aquaporin channel [41].

As shown by our experiments with permeabilized tumor cells, under certain conditions, NA(D)PH-dependent tumor cell dehydrogenases can support a free radical cascade initiated by the anthracyclines that culminates in the formation of the hydroxyl radical. We have previously shown that hydroxyl radical-induced DNA damage occurs after doxorubicin treatment in the clinic [15]. Further, we have also reported that a wide variety of hydroxyl radical trapping agents effectively protect intact Ehrlich tumor cells against the cytotoxicity of doxorubicin as assessed by soft agar cloning techniques [13]. Because the formation of formaldehyde from DMSO in these experiments required superoxide anion, hydrogen peroxide, and iron-EDTA, it is likely that the metal-catalyzed Haber-Weiss reaction was operating under our experimental conditions [42]. This is consistent with studies indicating that iron-EDTA chelate is an especially potent redox catalyst capable of stimulating a significant degree of ^·^OH production in the presence of hydrogen peroxide and a reducing agent [43, 44]. Under hypoxic conditions, the doxorubicin semiquinone may react directly with hydrogen peroxide to produce ^·^OH [45]; however, because our experiments were performed under highly aerobic conditions (agitation of vessels open to air in a shaking water bath), this is an unlikely explanation for the mechanism of ^·^OH formation in the studies presented here.

The importance of the oxidation-reduction cycle initiated by treatment of Ehrlich tumor cells with doxorubicin is related to the potent oxidizing power of various oxygen radical metabolites [46]. Thus, drug-induced oxygen radical formation could lead to the peroxidation of cellular phospholipids or the oxidation of critical sulfhydryl-containing enzymes and structural proteins with a subsequent loss of control of divalent cation transport or membrane integrity. Furthermore, since a potential role for oxygen radicals in certain forms of DNA damage is well-established, it is conceivable that free radical production by Ehrlich tumor cells after treatment with doxorubicin could also contribute to previously described effects of doxorubicin on nucleic acids [47]. One or more of these consequences of drug-related oxygen radical production could contribute to the tumoricidal effect of the anthracycline antibiotics.

In summary, we propose that drug-stimulated oxygen radical metabolism by the anthracycline antibiotics in multiple tumor cell compartments may contribute significantly to the antineoplastic activity of this class of drugs.

## Figures and Tables

**Table 1:** Kinetic constants for superoxide production by Ehrlich tumor subcellular fractions after treatment with doxorubicin. Superoxide production by tumor microsomes, mitochondria, and nuclei was assayed as described in Tables 2, 6, and 7. Kinetic constants were determined in triplicate to form the direct equation relating reaction velocity to substrate concentration as described in [48] using 8 different drug concentrations over a 10-fold concentration range.

Subcellular fraction	Superoxide formation	
	*K*_m_ (*μ*M)	*V*_max_ (nmol/min/mg)
Microsomes	124.9	22.6
Mitochondria	123.2	14.7
Nuclei	103.4	4.8

**Table 2:** Requirements for doxorubicin-stimulated superoxide formation in the tumor microsomal fraction. Superoxide production in tumor microsomes was determined in paired, 1 ml reaction mixtures which contained 150 mM potassium phosphate buffer, pH 7.4, 100 *μ*M EDTA, 56 *μ*M acetylated cytochrome c, 200 *μ*g of microsomal protein, and either 0 or 10 *μ*g of SOD. The chemotherapeutic agent was added to the paired reaction mixtures, where specified, before the initiation of the reaction by addition of NADPH (1 mM).

Experimental system	Superoxide production
	nmol cytochrome c reduced/min/mg
Control	0.51 ± 0.26 (3)^a^
-Microsomes	N.D. (3)^b^
-NADPH	N.D. (3)
-Cytochrome c	N.D. (3)
Using NADPH (100 *μ*M)	N.D. (3)
Using NADH (1 mM) rather than NADPH	0.97 ± 0.28 (3)
Doxorubicin (135 *μ*M)	14.71 ± 1.43 (6)^c^
-Microsomes	N.D. (3)^d^
-NADPH	N.D. (3)^d^
-Cytochrome c	N.D. (3)^d^
Using heat-denatured microsomes^e^	N.D. (3)^d^
Using heat-denatured SOD^e^	13.77 ± 0.37 (3)
Using NADPH (100 *μ*M)	10.07 ± 0.82 (6)
Using NADH (1 mM) rather than NADPH	2.81 ± 0.56 (3)^d^
Using NADP^+^ (1 mM) rather than NADPH	1.35 ± 0.26 (3)^d^
Using NAD^+^ (1 mM) rather than NADPH	0.36 ± 0.26 (3)^d^
Using FAD (1 mM)^f^ rather than NADPH	0.49 ± 0.49 (3)^d^
Using FMN (1 mM)^f^	N.D. (3)^d^
+DMSO (13 mM)	16.32 ± 0.51 (3)
+Catalase (1500 units)	14.36 ± 0.08 (3)
+Dicumarol (10 *μ*M)	14.41 ± 1.91 (3)

^a^Mean ± S.E.; number in parentheses is number of experiments performed; ^b^N.D. is not detectable; ^c^significantly different from control (*P* < 0.001); ^d^significantly different from complete system containing NADPH and doxorubicin (*P* < 0.01); ^e^microsomes or SOD heated for 60 min in a boiling water bath; samples containing heat-denatured SOD were paired against identical mixtures with native dismutase; ^f^FAD: flavin adenine dinucleotide; FMN: flavin mononucleotide.

**Table 3:** Effect of anthracycline antibiotics on superoxide production by the tumor microsomal fraction. Superoxide production in tumor microsomes was determined as described in Table 2. For these studies, all drugs were present at a concentration of 135 *μ*M.

Drug	Superoxide formation (nmol/min/mg)
Daunorubicin	10.00 ± 1.02 (5)^a,b^
Rubidazone	6.66 ± 0.84 (6)^b^
Aclacinomycin A	16.14 ± 0.85 (3)^b^
5-Iminodaunorubicin	0.45 ± 0.15 (3)

^a^Mean ± S.E.; number in parentheses is number of experiments performed; ^b^significantly higher than the control rate of superoxide formation in tumor microsomes (*P* < 0.01; Table 2).

**Table 4:** Effect of inhibitors of NADPH:cytochrome p-450 reductase on superoxide formation in tumor microsomes. NADPH:cytochrome P-450 reductase was assayed at 30°C as described in Materials and Methods using nonacetylated cytochrome c and 200 *μ*g of microsomal protein per ml. Reactions were initiated with 100 nmol of NADPH. Where indicated, the reaction mixtures were preincubated with DTNB or NADP^+^ for 2 min prior to the initiation of cytochrome c reduction. In these experiments, superoxide production was assessed as described in Table 2 except that the NADPH concentration was 100 *μ*M rather than 1 mM.

Experimental system	NADPH:cytochrome P-450 reductase activity (nmol/min/mg)	Superoxide production (nmol/min/mg)
Control	233.5 ± 12.5 (3)^a^	
+DTNB (100 *μ*M)	54.1 ± 1.6 (3)^b^	
+NADP^+^ (1 mM)	64.6 ± 1.5 (3)^b^	
Doxorubicin (135 *μ*M)		10.1 ± 1.0 (3)
+DTNB (100 *μ*M)		4.2 ± 0.4 (3)^b^
+NADP^+^ (1 mM)		2.7 ± 0.2 (3)^b^

^a^Mean ± S.E.; numbers in parentheses are numbers of experiments; ^b^significantly different from samples without inhibitor present (*P* < 0.01).

**Table 5:** Antioxidant enzyme levels in tumor subcellular fractions. Ehrlich tumor microsomes, mitochondria, and nuclei were prepared and assayed for glutathione peroxidase and SOD activity as described in Materials and Methods. The “cytosol” fraction was the supernatant from the final 65,000 x *g* centrifugation step used in the preparation of the microsomal fraction. Before determination of enzyme activity, the mitochondrial fraction was exposed to ultrasonic disruption on ice with 4 bursts of 15 sec each at 90 watts output to eliminate permeability barriers to appropriate substrates in these assays. The supernatant and pellet resulting from centrifugation of the sonicated mitochondria at 105,000 x *g* and 4°C for 60 min were also assayed from enzyme activities.

Tumor cell fraction	Glutathione peroxidase (nmol/min/mg)	SOD (*μ*g SOD/mg)
Cytosol	211.6 ± 11.0 (4)^a^	8.1 ± 0.8 (3)
Microsomes	22.5 ± 1.6 (4)	1.4 ± 0.3 (3)
Nuclei	10.1 ± 2.2 (3)	0.5 ± 0.1 (3)
Mitochondria	55.5 ± 8.0 (3)	5.6 ± 0.3 (3)
105,000 x *g* supernatant	197.9 ± 20.9 (3)	3.2 ± 0.4 (3)
105,000 x *g* pellet	N.D. (3)^b^	1.6 ± 0.2 (3)

^a^Mean ± S.E.; numbers in parentheses are numbers of experiments; ^b^N.D. is not detectable.

**Table 6:** Requirements for anthracycline-enhanced superoxide anion production by the tumor mitochondrial fraction. Superoxide formation in the tumor mitochondrial fraction was examined using paired, 1 ml reaction mixtures containing 250 mM sucrose, 20 mM HEPES, pH 8.2, 100 *μ*M EDTA, 56 *μ*M acetylated cytochrome c 100 *μ*g of mitochondrial protein, and either 0 or 10 *μ*g of SOD. The reaction mixture was preincubated for 5 min at 37° with 4 *μ*M rotenone before initiation of the reaction with 100 *μ*M NADH.

Reaction mixture	Superoxide production (nmol/min/mg)
Control	1.12 ± 0.15 (7)^a^
-NADH	N.D. (3)^b^
-Mitochondrial fraction	N.D. (3)
-Rotenone	1.02 ± 0.01 (3)
Using NADPH (100 *μ*M) rather than NADH	0.77 ± 0.26 (3)
Doxorubicin (135 *μ*M)	7.29 ± 0.61 (10)^c^
-NADH	0.97 ± 0.05 (3)^d^
-Mitochondrial fraction	0.61 ± 0.20 (3)^d^
Using heat-denatured mitochondria	1.28 ± 0.56 (3)^d^
-Rotenone	5.00 ± 0.36 (3)^d^
Using NADPH (100 *μ*M) rather than NADH	2.76 ± 0.44 (3)^d^
-Acetylated cytochrome c	N.D. (3)^d^
-EDTA	5.51 ± 1.22 (3)
Using heat-denatured SOD	5.10 ± 0.61 (3)
Daunorubicin (135 *μ*M)	6.38 ± 1.63 (3)^c^
Rubidazone (135 *μ*M)	5.71 ± 1.02 (3)^c^
Aclacinomycin A (135 *μ*M)	3.32 ± 0.31 (3)^c^
5-Iminodaunorubicin (135 *μ*M)	0.51 ± 0.15 (3)

^a^Mean ± S.E.; numbers in parentheses are numbers of experiments; ^b^N.D. is not detectable; ^c^significantly different from control (*P* < 0.01); ^d^significantly different from complete system containing doxorubicin alone (*P* < 0.01).

**Table 7:** Requirements for anthracycline-stimulated superoxide formation by the nuclear fraction. Superoxide formation by tumor nuclei was examined using paired 1 ml reaction mixtures containing 250 mM sucrose, 20 mM HEPES, pH 7.4, 100 *μ*M EDTA, 56 *μ*M acetylated cytochrome c, 200 *μ*g of nuclear protein, and either 0 or 10 *μ*g of SOD. The reaction was carried out at 37°C and was initiated by the addition of 1 mM NADPH after the chemotherapeutic agent was added.

Reaction mixture	Superoxide formation (nmol/min/mg)
Control	0.36 ± 0.05 (7)^a^
Using NADH (1 mM) rather than NADPH	0.28 ± 0.08 (3)
Doxorubicin (135 *μ*M)	3.29 ± 0.33 (12)^b^
-NADPH	N.D. (3)^c,d^
-Acetylated cytochrome c	N.D. (3)^d^
Using heat-denatured nuclei	N.D. (3)^d^
Using NADH (1 mM) rather than NADPH	0.31 ± 0.15 (3)^d^
+Heat-denatured SOD	2.73 ± 0.31 (3)
+DMSO (13 mM)	4.21 ± 0.13 (3)
+Catalase (1500 units/ml)	3.70 ± 0.13 (3)
Using FAD (1 mM) rather than NADPH	N.D. (3)^d^
Using FMN (1 mM) rather than NADPH	N.D. (3)^d^
Daunorubicin (135 *μ*M)	6.63 ± 0.64 (3)^b^
Rubidazone (135 *μ*M)	3.01 ± 0.31 (3)^b^
Aclacinomycin A (135 *μ*M)	5.53 ± 0.38 (3)^b^

^a^Mean ± S.E.; numbers in parentheses are numbers of experiments; ^b^significantly different from control (*P* < 0.01); ^c^N.D. is not detectable; ^d^significantly different from complete system containing doxorubicin alone (*P* < 0.01).

**Table 8:** Effect of anthracycline antibiotics on oxygen consumption by Ehrlich tumor cells. Oxygen consumption by Ehrlich cells was examined at 37°C as described in Materials and Methods; the total 3 ml volume contained 1.5 × 10^7^ tumor cells and the final KCN concentration, where used, was 2 mM.

Reaction system	Oxygen consumption (nmol O_2_/min/5 × 10^6^ cells)	
	+KCN	-KCN
Control	0.54 ± 0.04^a^	9.37 ± 0.52
+BCNU (100 *μ*g/ml)	0.58 ± 0.08	9.56 ± 0.80
+Glucose (10 mM)	0.58 ± 0.10	9.67 ± 0.90
+Antimycin A (10 *μ*g/ml) replacing KCN	0.50 ± 0.10	
-Cells	N.D.^b^	
Doxorubicin (90 *μ*M)	0.86 ± 0.02^c^	10.00 ± 1.20
Doxorubicin (200 *μ*M)	1.04 ± 0.12^c^	11.46 ± 1.18
Doxorubicin (400 *μ*M)	1.13 ± 0.04^c^	8.82 ± 0.22
+BCNU (100 *μ*g/ml)	1.39 ± 0.08^d^	—
+Adenosine (1 mM)	1.06 ± 0.04	—
+Glucose (10 mM)	1.61 ± 0.08^d^	9.47 ± 0.16
+Antimycin A (10 *μ*g/ml) replacing KCN	1.19 ± 0.04^c^	—
-Cells	N.D.	—
Doxorubicin (1 mM)	4.54 ± 0.40^c^	—
Daunorubicin (400 *μ*M)	1.39 ± 0.06^c^	—
5-Iminodaunorubicin (400 *μ*M)	0.64 ± 0.06	—

^a^Mean ± S.E. of 3 to 15 experiments; ^b^N.D. is not detectable; ^c^significantly different from control, at *P* < 0.01; ^d^significantly different from samples containing doxorubicin alone, at *P* < 0.01.

**Table 9:** Requirements for doxorubicin-stimulated hydroxyl radical formation by detergent-treated Ehrlich carcinoma cells. Hydroxyl radical production by Ehrlich carcinoma cells was determined by measurements of formaldehyde production from DMSO; the standard reaction mixture contained 100 mM DMSO, 100 *μ*M EDTA, 50 *μ*M FeS0_4_, 1 mM NADPH, 0.1% Triton X-100, 10^7^ tumor cells/ml, and the indicated concentration of doxorubicin in a final volume of 7 ml of PBS at pH 7.2. Data are expressed as the mean ± S.E. of formaldehyde production for the 2 hr reaction interval in each experimental group; the total number of experiments for each group (*n*) is given in parentheses.

Experimental system	Hydroxyl radical production (nmol formaldehyde/10^7^ cells)
Control	0.0 ± 0.0 (*n* = 5)^a^
Doxorubicin (250 *μ*M)	71.4 ± 10.4 (*n* = 15)^b^
Minus cells	0.0 ± 0.0 (*n* = 3)^c^
Heat denatured cells^d^	0.0 ± 0.0 (*n* = 3)^c^
Minus DMSO	0.0 ± 0.0 (*n* = 3)^c^
Minus EDTA	0.0 ± 0.0 (*n* = 3)^c^
Minus FeS0_4_	0.0 ± 0.0 (*n* = 3)^c^
Using 1 *μ*M rather than 50 *μ*M FeS0_4_	13.2 ± 4.6 (*n* = 3)^c^
Using FeC13 (50 *μ*M) rather than FeS0_4_	30.9 ± 10.0 (*n* = 3)^b^
Minus NADPH	0.0 ± 0.0 (*n* = 3)^c^
Minus NADPH plus NADH (1 mM)	30.6 ± 8.3 (*n* = 5)^e^
Minus NADPH plus succinate (5 mM)	0.0 ± 0.0 (*n* = 3)^c^
Minus Triton X-100	0.0 ± 0.0 (*n* = 3)^c^

^a^Mean ± S.E.; ^b^significantly different from the control group (*P* < 0.001); ^c^significantly different from complete reaction mixture containing doxorubicin and 50 *μ*M FeS0_4_ (*P* < 0.001); ^d^tumor cells autoclaved for 60 min; ^e^significantly different from control and from complete reaction mixture containing NADPH (*P* < 0.01).

**Table 10:** Effect of oxygen radical scavengers on doxorubicin-enhanced formaldehyde production by detergent-treated Ehrlich tumor cells. Hydroxyl radical production by Ehrlich carcinoma cells was determined exactly as described in Table 9; data have been expressed as the mean ± S.E. of formaldehyde production for the 2 hr reaction interval in each experimental group, and the total number of experiments (*n*) has been given in parentheses.

Experimental conditions	Formaldehyde production (nmol/10^7^ cells)
Doxorubicin (250 *μ*M)	60.4 ± 5.6 (6)^a^
Plus SOD (20 *μ*g/ml)	17.0 ± 9.6 (6)^b^
Plus heat-denatured SOD (20 *μ*g/ml)^c^	49.1 ± 5.0 (3)
Plus catalase (3000 units/ml)	0.0 ± 0.0 (3)^d^
Plus heat-denatured catalase (3000 units/ml)	44.3 ± 7.5 (3)
Plus sodium benzoate (100 mM)	34.3 ± 3.4 (3)^b^
Plus mannitol	
100 mM	41.7 ± 3.5 (3)^b^
200 mM	23.6 ± 3.5 (3)^d^
Plus diethylurea (100 mM)	25.8 ± 6.0 (3)^b^
Plus dimethylthiourea (100 mM)	0.0 ± 0.0 (3)^d^
Plus urea (100 mM)	50.9 ± 9.6 (3)

^a^Mean ± S.E.; ^b^significantly different from samples treated with doxorubicin alone (*P* < 0.01); ^c^SOD and catalase were autoclaved for 60 min; ^d^significantly different from samples treated with doxorubicin alone (*P* < 0.001).

## Data Availability

The data used to support the findings of this study are included within the article.

## Abbreviations

EGTA: Ethylene-glycol-bis(*β*-amino-ethylether)-N, N^1^-tetraacetic acid
HEPES: N-2-Hydroxyethylpiperazine-N^1^-2-ethanesulfonic acid
DTNB: 5-5′-Dithiobis(2-nitrobenzoic acid)
SOD: Superoxide dismutase
PBS: Dulbecco's phosphate buffered saline
BCNU: 1,3-Bis-chloro(2-chloroethyl)-1-nitrosourea
^·^OH: Hydroxyl radical
ROS: Reactive oxygen species.

## Supplementary Materials

Supplementary Materials Supplementary Table 1: effect of doxorubicin on oxygen consumption by the tumor microsomal fraction. Supplementary Table 2: effect of doxorubicin on oxygen consumption by the tumor mitochondrial fraction. Supplementary Table 3: effect of alterations in NADPH:cytochrome p-450 reductase level on superoxide formation by tumor nuclei.
