# Supplementary material for: Mechanisms of Anthracycline-Enhanced Reactive Oxygen Metabolism in Tumor Cells
Source: Oxid Med Cell Longev. 2019 Dec 3;2019:9474823. doi: 10.1155/2019/9474823 (PMC6914999; doi:10.1155/2019/9474823)
Supplement: Supplementary Materials — Supplementary Table 1: effect of doxorubicin on oxygen consumption by the tumor microsomal fraction. Supplementary Table 2: effect of doxorubicin on oxygen consumption by the tumor mitochondrial fraction. Supplementary Table 3: effect of alterations in NADPH:cytochrome p-450 reductase level on superoxide formation by tumor nuclei. [file 9474823.f1.docx]

**Supplementary materials**

Supplementary Table 1: Effect of doxorubicin on oxygen consumption by the tumor microsomal fraction. Oxygen consumption in tumor microsomes was examined as described in “Materials and Methods” using 150 mM potassium phosphate buffer, pH 7.4, 1 mM NADPH, and 500 µg/ml microsomal protein. For these experiments doxorubicin and other reactants were equilibrated in the 3-ml reaction vessel for 4 min, and then oxygen consumption was initiated by the addition of NADPH.

Oxygen consumption

Reaction system (nmol 0_2_/min/mg)

Control 1.54 ± 0.32 (6)^a^

+ EDTA (100 µM) 1.23 ± 0.15 (4)

- NADPH N.D. (3)^b^

Doxorubicin (135 µM) 10.87 ± 0.56 (11)^c^

+ EDTA (100 µM) 11.58 ± 0.76 (6)^c^

- NADPH N.D. (3)^d^

- microsomes N.D. (3)^d^

+ acetylated cytochrome c (56 µM) 3.70 ± 0.12 (3)^d^

+ KCN (1 mM) 14.73 ± 0.92 (3)^d^

+ Azide (1 mM) 10.83 ± 0.12 (4)

+ dicumarol (10 µM) 12.46 ± 0.24 (3)

^a^  Mean ± S.E.; numbers in parentheses are numbers of experiments.

^b^ N.D. is not detectable.

^c^ Significantly different from control (*P* < 0.001).

^d^ Significantly different from complete system containing doxorubicin alone, (*P* < 0.01).

Supplementary Table 2: Effect of doxorubicin on oxygen consumption by the tumor mitochondrial fraction. Oxygen consumption in tumor mitochondria was examined as described in “Materials and Methods” using 250 mM sucrose, 20 mM HEPES, pH 8.2, 100 µM EDTA, and 200 µg of mitochondrial protein. The 3-ml reaction mixture was preincubated with 4 µM rotenone and doxorubicin for 5 min at 37º before initiation with 1 mM NADH.

Oxygen consumption

Reaction system (nmol O_2_/min/mg)

Control 5.57 ± 0.60 (7)^a^

- NADH N.D. (3)^b^

Doxorubicin (135 µM) 10.95 ± 0.60 (7)^c^

- NADH N.D. (3)

+ KCN (1 mM) 9.06 ± 0.50 (4)^c^

+ Azide (1 mM) 9.25 ± 0.80 (3)^c^

+ Dicumarol (10 µM) 10.65 ± 0.30 (3)^c^

+ Acetylated cytochrome c (56 µM) 6.27 ± 0.30 (3)^d^

^a^ Mean ± S.E.; numbers in parentheses are numbers of experiments.

^b^N.D. is not detectable.

^c^Significantly different from control (*P* < 0.01).

^d^Significantly different from complete system containing doxorubicin alone, (*P* < 0.01).

Supplementary Table 3: Effect of alterations in NADPH:cytochrome p-450 reductase level on superoxide formation by tumor nuclei. NADPH:cytochrome p-450 reductase was assayed exactly as described in Table 4 using 200 µg of nuclear protein per ml. Reactions were initiated by addition of 100 µM NADPH. Superoxide was measured with 100 µM NADPH as described in Table 6. Where indicated, 0.1% (vol/vol) Triton X-100 was added to the nuclear suspension prior to the final Dounce homogenization and subsequent sucrose gradient centrifugation.

| *Experimental System* | *NADPH: cytochrome P-450 reductase activity*  *(nmol/min/mg)* | | *Superoxide production*  *(nmol/min/mg)* | |
| --- | --- | --- | --- | --- |
|  | +Triton | -Triton | +Triton | -Triton |
| Control | 0.10 ± 0.05 (3)^a,b^ | 5.28 ± 0.51 (3) | 0.38 ± 0.05 (3) | 0.34 ± 0.10 (3) |
| +NADP^+^ (1 mM) |  | 1.48 ± 0.05 (3)^c^ |  |  |
|  |  |  |  |  |
| Doxorubicin (135 µM) |  |  | 0.46 ± 0.05 (3)^b^ | 2.96 ± 0.08 (3) |
| +NADP^+^ (1 mM) |  |  |  | N.D. (3)^d^ |

^a^ Mean ± S.E.; numbers in parentheses are numbers of experiments.

^b^Significantly different from corresponding sample without Triton X-100 (*P* < 0.01).

^c^Significantly different from sample without NADP^+^ (*P* < 0.01).

^d^N.D. is not detectable.
